# Skilled musicians are not subject to the McGurk effect

**DOI:** 10.1038/srep30423

**Published:** 2016-07-25

**Authors:** Alice M. Proverbio, Gemma Massetti, Ezia Rizzi, Alberto Zani

**Affiliations:** 1 Milan-Mi Center for Neuroscience, Dept. of Psychology, University of Milano-Bicocca, Milan, Italy; 2 IBFM-CNR, Institute of Bioimaging and Molecular Physiology, National Research Council, Milan, Italy

## Abstract

The McGurk effect is a compelling illusion in which humans auditorily perceive mismatched audiovisual speech as a completely different syllable. In this study evidences are provided that professional musicians are not subject to this illusion, possibly because of their finer auditory or attentional abilities. 80 healthy age-matched graduate students volunteered to the study. 40 were musicians of Brescia *Luca Marenzio Conservatory of Music* with at least 8–13 years of musical academic studies. /la/, /da/, /ta/, /ga/, /ka/, /na/, /ba/, /pa/ phonemes were presented to participants in audiovisual congruent and incongruent conditions, or in unimodal (only visual or only auditory) conditions while engaged in syllable recognition tasks. Overall musicians showed no significant McGurk effect for any of the phonemes. Controls showed a marked McGurk effect for several phonemes (including alveolar-nasal, velar-occlusive and bilabial ones). The results indicate that the early and intensive musical training might affect the way the auditory cortex process phonetic information.

This phonetic illusion occurring during speech perception was first reported by McGurk and MacDonald^1^, who found that when viewing edited movie clips of an actor articulating one syllable in synchronization with the soundtrack of other syllables, individuals often perceive a syllable incongruent with either the visual or auditory input. For example, a visual /ga/ with an auditory /ba/ may be perceived as /da/; and a visual /ka/ with an auditory /pa/ elicits the perception of /ta/2.

A large inter-subject variability has been found in the strength of this illusion across groups. For example, reduced McGurk effects have been found in people with one eye^3^, possibly because of the reduced impact of the interfering incongruent visual information. Individuals with schizophrenia were observed to exhibit illusory perception less frequently than healthy controls, despite non-significant differences in perceptual performance during control conditions^4^. Setti and coworkers^5^ found increased illusory perception in a sample of older adults (mean age: 65 years) compared with younger adults (mean age: 22 years) and interpreted this increase in terms of perceptual decline in the older group. Furthermore, healthy adolescents seem to experience the illusion more frequently than healthy adults^6^, which suggests a refinement of their phonetic perception during brain development (e.g.)^7^.

In an interesting fMRI study it was investigated the neural underpinnings of inter-individual variability in the perception of the McGurk illusion^8^. The amplitude of the response in the left STS was significantly correlated with the likelihood of perceiving the McGurk effect: a weak STS response meant that a subject was less likely to perceive the McGurk effect, while a strong response meant that a subject was more likely to perceive it. These results suggest that the left STS is a key locus for explaining inter-individual differences in speech perception.

The aim of the present study was multifold. First of all, we wished to investigate in greater depth the existence of the McGurk illusion for the Italian language, by using a large variety of phonemes characterized by different articulatory mechanics and places of articulation. Indeed only a few studies have explored the existence of the McGurk illusion in Italian speakers so far. Gentilucci and Cattaneo^9^ investigated audiovisual integration processes relative to the perception of 3 non-sense strings (ABA, AGA, ADA), while D’Ausilio *et al*.^10^ considered only /ba/, /ga/, /pa/, /ka/ phonemes. Bovo and colleagues^11^ performed a more systematic study by presenting 8 phonemes to 10 Italian participants. Overall, all studies found that the McGurk illusion in Italian speakers is comparable to what found for other languages^2^.

Secondly, we wished to determine whether professional musicians might be more resistant towards the McGurk illusion, in the hypothesis that they had developed finer acoustic abilities to ignore the incongruent labial information. No similar study (in our knowledge) has ever been conducted, in any language.

Overall, it has been clearly shown that extensive musical training has powerful effects on many cerebral domains^12^,^13^. These differences include motor performance (e.g.)^14^,^15^,^16^,^17^, music reading and note coding^18^, visuomotor transformation^19^,^20^, callosal inter-hemispheric transfer^21^,^22^, sound and speech processing^23^,^24^,^25^,^26^,^27^,^28^,^29^,^30^,^31^,^32^, audiomotor integration^33^,^34^,^35^,^36^,^37^. Musicians are obviously particularly skilled in auditory analysis and this might have long lasting effects on speech processing ability^38^.

Furthermore, evidences supporting differences between musicians and non-musicians in the functionality related to audiovisual integration have been provided for example by Lee and coworkers^39^, and Paraskevopoulos and coworkers^40^.

In this framework we aimed at investigating whether the benefits linked to being a musician included a stronger resistance to degraded speech information and a reduced/absent susceptibility to the McGurk illusion.

One possibility is that musicians focused their attention more to the auditory than the visual modality, thus reducing the McGurk illusion. In the typical McGurk paradigm if the audiovisual information is incongruent listeners may decide to report what they see or what they hear. And indeed Massaro and other researchers^41^,^42^,^43^,^44^,^45^ have shown that the strength of the McGurk illusion increased as the percentage of responses based on the acoustic component decreased. At this regard, Gurler *et al*.^46^ have shown a correlation between lips fixation and susceptibility to the McGurk illusion. By means of infrared eye tracker monitoring they were able to record where the subjects tended to fixate the speakers (on the eyes vs. the mouth) during audiovisual perception. It was found that those who stared longer to the mouth had a more accurate perception of the syllable mimed in the video, thus increasing the perception of incongruity between the auditory and visual stimulation, and causing a significant increase in the probability of occurrence of the McGurk illusion. To avoid this bias, in the present study we specifically asked participants to report what they heard (acoustically). In addition we located a fixation point on the tip of the nose of the actors, in order to avoid changes in fixation during phoneme processing and saccades that might fall on the lips.

In this study, 80 healthy, age-matched, graduate male and female volunteers were tested both in multimodal (McGurk condition) and unimodal (only auditory and only visual) conditions.

## Results

**Figure 1:**
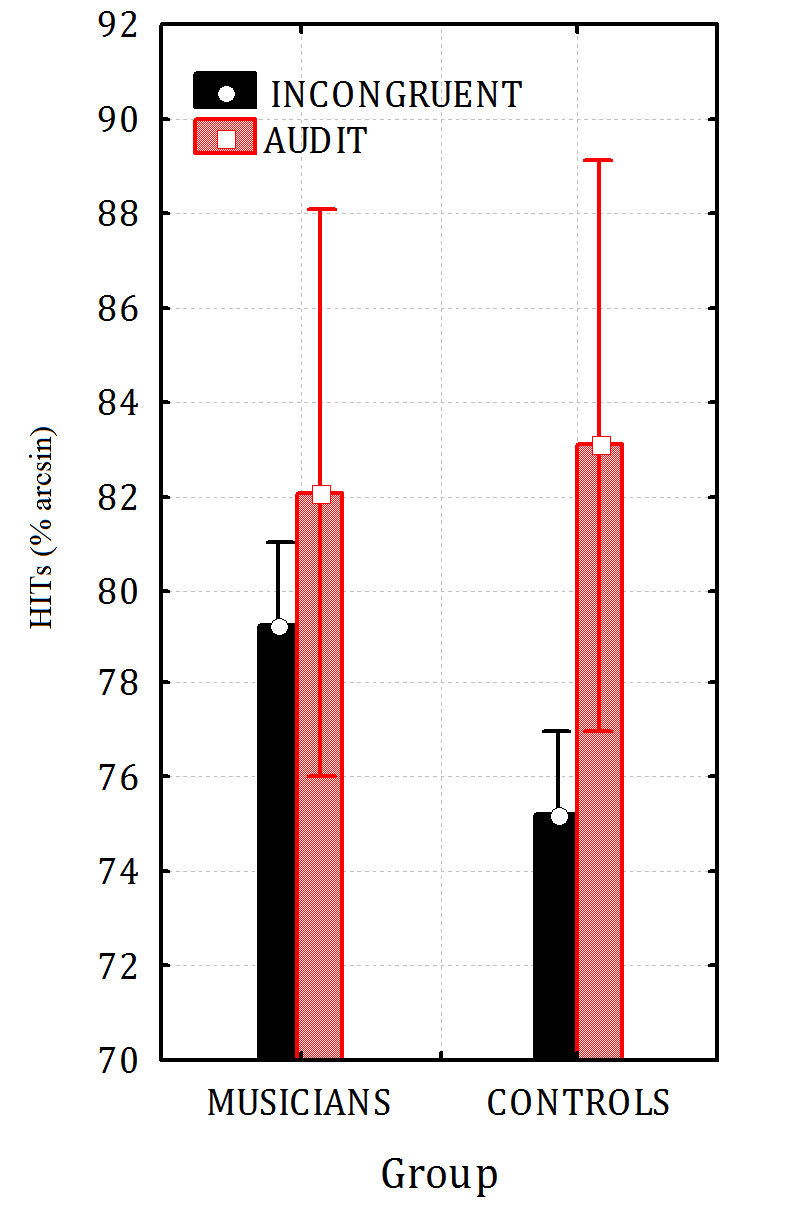
Hits percentages recorded in the incongruent multimodal vs. auditory condition in musicians and controls as a function of stimulus congruence. Effect of Musicianship in the lack of McGurk illusion (p < 0.033).

No difference between the groups was found in the ability to recognize phonemes in the various non-mismatched stimulation conditions. Overall a significant difference between tasks was found with a very low performance in the visual task and no difference between the auditory unimodal and audiovisual congruent conditions (Musicians: visual = 10%; auditory = 90%; audiovisual = 93.75%. Controls: visual = 11.25%; auditory = 93.75%; audiovisual = 95%) as demonstrated by Wilcoxon tests applied to row percentages of correct recognitions (Musicians: visual vs. audit. p = 0.0278; visual vs. audiovisual p = 0.0278; Controls: visual vs. audit. p = 0.0117; visual vs. audiovisual p = 0.0117; Musicians vs. controls = n.s.; Audiovisual vs. auditory = n.s.). The mixed model ANOVA performed on hit percentages recorded across groups and conditions (auditory vs incongruent audiovisual condition, collapsed across phonemes), gave rise to the significance of group factor (F2,14 = 6.3, p = 0.03), with musicians showing a better performance than controls (see Fig. 1 for arcsin transformed means and standard deviations). The effect of condition was also significant (F2,14 = 5.0, p < 0.0041) with higher hit rates for auditory (82.56%, SE = 5.11) than incongruent audiovisual stimuli (77.22%, SE = 1.49). No interaction between group and condition was found for this contrast.

**Figure 2:**
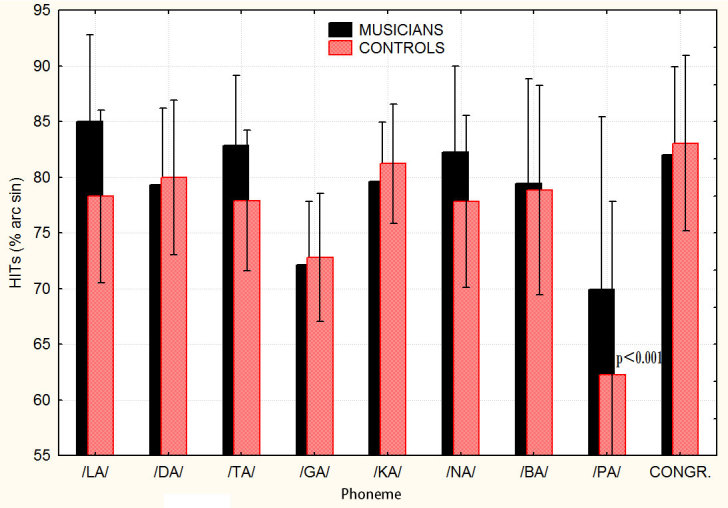
Correct phoneme recognitions (%) as a function of the incongruent auditory information, and as compared to the congruent condition, for the two groups of participants in the McGurk experiment. The dependent variable was the performance (arsine transformed hit percentages) as a function of the phonemes auditorily perceived, that is the audio. This analysis was able to show how phoneme recognition was differentially affected by each of the incongruent phonemes presented.

Two further repeated measures ANOVAs were performed for the 2 groups to investigate in detail the effect of lip movements (visual) or phonetic (auditory) incongruent information in the McGurk experiment. The analyses of data performed as a function of the phonemes auditorily perceived showed the statistical significance of “condition” factor (congruent vs. incongruent conditions) (F 8,112 = 3.646; p < 0.0008), but only for controls. The analysis of group effects and Tukey post-hoc tests showed no significant difference between the congruent and the McGurk conditions in musicians but only in controls, especially for /PA/ stimuli, as can be appreciated in Fig. 2.

**Figure 3:**
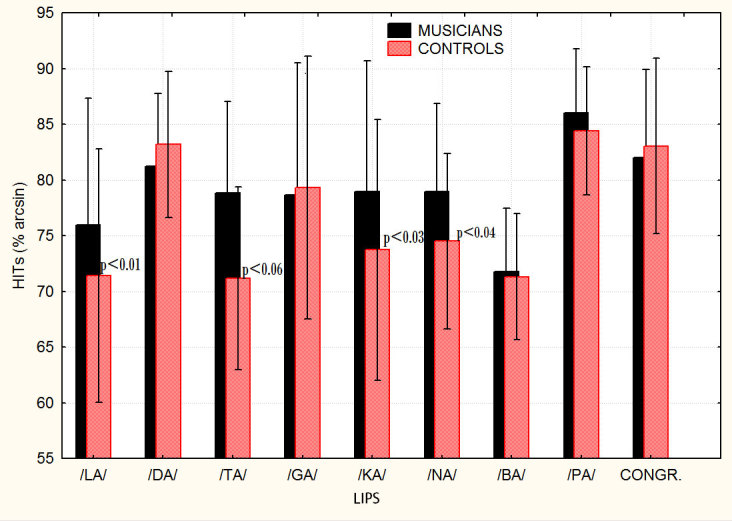
Correct phoneme recognitions (%) as a function of the incongruent labial information, and as compared to the congruent condition, for the two groups of participants in the McGurk experiment. The dependent variable was the performance (arsine transformed hit percentages) as a function of the lip movements visually perceived. This analysis was able to show how phoneme recognition was differentially affected by each of the incongruent lip movement (labial) visually shown.

Similarly, the “condition” factor (congruent vs. incongruent conditions) as a function of the labial (lip movements) perceived was statistically significant (F 8,112 = 2.685; p < 0.0097), but only for controls. Post-hoc comparisons showed significant differences between the congruent and the illusory conditions for /LA/ p = 0.018, /KA/ p = 0.03, /NA/ p = 0.04, with a tendency to significance for /TA/ p = 0.06 and /BA/ p = 0.07, in controls. Conversely, no significant decrease in performance between the congruent and the incongruent McGurk conditions was found in musicians (see Fig. 3).

## Discussion

The aims of the study were: 1) to investigate in greater depth the existence of the McGurk illusion for the Italian language, by using a large variety of phonemes characterized by different articulatory mechanics and places of articulation, 2) to determine whether professional musicians might be more resistant towards the McGurk illusion, in the hypothesis that they had developed finer acoustic abilities to ignore the incongruent labial information. The results showed a lack of McGurk effect in musicians: while the latter were not subject to interferences due to audiovisual conflicts, controls reported consistent McGurk illusions in the incongruent condition, especially for velar occlusive, dental, nasal and bilabial phonemes.

Overall the data show that all participants were unable to correctly recognize phoneme solely on the basis of labial information (10–11% of hits for mute videos). This piece of data agrees with available literature showing a modest performance in isolated phoneme identification in normal-hearing lipreaders (between 6 and 18%), and a higher (but still poor) performance in deaf lipreaders between (21 and 43%)^47^.

Although the existence of a visual speech area named TVSA (*temporal visual speech area*), located posteriorly and ventrally to the multisensory pSTS (posterior superior temporal sulcus), has been clearly demonstrated)^48^,^49^,^50^,^51^ the activation of this area alone is not sufficient to allow speech recognition in untrained hearing speakers. Massaro suggested that, “because of the data-limited property of visible speech in comparison to audible speech, many phonemes are virtually indistinguishable by sight, even from a natural face, and so are expected to be easily confused” 52.

Our data also show a lack of difference between the unimodal auditory condition (listening to syllables) and the congruent audiovisual condition (watching and listening), probably because phonemes were well perceivable, the environment was silent and without distractions. Indeed it seems that perception of speech is improved only when presentation of the degraded audio signal is accompanied by concordant visual speech gesture information^53^,^54^.

Overall, musicians were much better than controls in recognizing phonemes in incongruent audiovisual conditions as compared to the auditory condition, as demonstrated by the mixed anova. Indeed, while musicians were not subject to interferences due to audiovisual conflicts, controls reported consistent McGurk illusions in the incongruent condition. In this group, perception of congruent (vs. incongruent) tongue movements facilitated auditory speech identification^10^ in the multimodal McGurk condition It has been shown that, when auditory and visual speech are presented simultaneously information converges early in the stream of processing. As a result, it may happen that an incongruent visual stimulation interfere with auditory recognition and altered auditory perception could arise to conflict resolution with incongruent auditory inputs. This phenomena is thought to contribute to the McGurk illusion. Primary auditory cortex activation by visual speech has been demonstrated^55^,^56^ while other studies have proofed the existence of multimodal audiovisual neurons in the STS engaged in the synthesis of auditory and visual speech information^55^,^57^.

The analysis of the effects of audiovisual incongruence shows that recognition errors were very frequent in controls for velar occlusive, dental, nasal and bilabial phonemes, independent of audiovisual combination, but participants seemed to be more accurate when bilabials were paired with another bilabial (see Table 1 and 2 for a full report of qualitative results). This pattern of results is in strong agreement with the findings by D’Ausilio *et al*.^10^ or Bovo and coworkers^11^. The latter investigated the McGurk illusion in ten (non-musician) Italian speakers by presenting /ba/, /da/, /ga/, /pa/, /ta/, /ka/, /ma/, /na/ phonemes, coherently or incoherently dubbed. Stronger McGurk illusions were found when bilabial phonemes were presented acoustically and non-labials (especially alveolar-nasal and velar-occlusive phonemes) visually.

Our data show that skilled musicians with at least 8–13 years of academic studies are not subject to the McGurk illusion. This might be due to their finer acoustic/phonemic processing^58^ or enhanced neural representation of speech when presented in acoustically-compromised conditions^59^,^60^,^61^. Strait and Kraus^31^ have shown that music training improves speech-in-noise perception. In an interesting ERP study Zendel *et al*.^62^ not only showed that encoding of speech in noise was more robust in musicians than in controls, but that there was a rightward shift of the sources contributing to the N400 as the level of background noise increased. Moreover, the shift in sources suggests that musicians, to a greater extent than nonmusicians, may increasingly rely on acoustic cues to understand speech in noise.

At this regard it can be hypothesized that the lesser susceptibility of musicians to the McGurk illusion is related to a different pattern of functional specialization of auditory, and speech processing brain areas. Specifically, with regard to basic audiovisual integration, differences between musicians and non-musicians have been demonstrated. Existing evidence indicates a greater contribution of the connectivity of the left Broca area in musicians for audiovisual tasks, which directly links to the processing of speech. For example, Paraskevopoulos and coauthors^63^ investigated the functional network underpinning audiovisual integration via MEG recordings and found a greater connectivity in musicians than nonmusicians between distributed cortical areas, including a greater contribution of the right temporal cortex for multisensory integration and the left inferior frontal cortex for identifying abstract audiovisual incongruences.

Several other studies suggest that the linguistic brain and the STS might be less left lateralized in musicians than non-musicians, in favor of an involvement of the right homologous counterpart. For example Parkinson *et al*.^64^ found enhanced connectivity relating to pitch identification in the right superior temporal gyrus (STG) of musicians. Again, Lotze *et al*.^65^ found a higher activity of the right primary auditory cortex during music execution in amateurs vs. professional musicians that may reflect an increased strength of audio-motor associative connectivity. Indeed, it has been shown that the left STS is more active in people more susceptible to the illusion (as compared to less susceptible individuals) during McGurk perception of incongruent audiovisual phonetic information, both in adults^66^ and in children^67^. In Nath and Beaucham^66^ study the amplitude of the response in the left STS was significantly correlated with the likelihood of perceiving the McGurk effect: a weak lSTS response meant that a subject was less likely to perceive the McGurk effect, while a strong response meant that a subject was more likely to perceive it. Furthermore, the McGurk is illusion is disrupted upon stimulation of the left STS via transcranial magnetic stimulation^68^ in a narrow temporal window from 100 ms before auditory syllable onset to 100 ms after onset.

Finally, the present data showed that two groups of musicians and controls did not differ in their ability to recognize the phonemes in any of the (congruent) conditions. This suggests that music training did not affect syllable comprehension per se, in not-degraded and not noisy circumstances, nor that the two groups differed in their basic auditory, visual, or acoustical/verbal ability. The lack of difference in the auditory condition might be also explained by either a ceiling effect, or the fact that the effect of musical training is observed when a complex auditory processing is required (for example pitch discrimination^69^,^70^. This is indicated for example by MMN studies showing a significant difference between musicians and non-musicians in the brain response to deviant stimuli belonging to tonal patterns^71^ or melodies^72^ as opposed to a lack of group differences for processing single tones^72^.

Overall, the lack of McGurk illusion in musicians might be interpreted in terms of the effect of music training on adaptive plasticity in speech-processing networks as proposed by Patel^38^. In his OPERA theoretical model Platel suggested that one reason why musical training might benefit the neural encoding of speech is because there is a certain anatomical overlap in the brain networks that process acoustic features used in both music and speech (e.g., waveform periodicity, amplitude envelope). Since during noisy condition musicians seemed to rely more on acoustic (than phonetic) inputs, this might explain the reduced effect of inconsistent signals coming from the left visual speech area (TVSA) or left audiovisual STS neurons. However, the present study did not use neuroimaging techniques to investigate the neural mechanisms underlying the McGurk illusion, therefore the hypotheses presented remain speculative and deserve further experimentation.

In the end it cannot be excluded that the lack of McGurk effect in musicians might be in part due to their stronger ability to focus attention on the auditory modality^38^. But in order to prevent this all participants were specifically instructed to report what they had heard (regardless of what they had seen). Furthermore a fixation point was located on the tip of the nose of speakers, in order to avoid changes in fixation and saccades that might fall on the lips, thus increasing the McGurk illusion^46^. However, in this study, participant ocular movements were not directly monitored, as it would have been made possible by the use of an eye tracking system.

## Methods

### Participants

40 right-handed musicians (15 females, 35 males) aging on average 23.4 years (see Table 3 for details on musician participants) took part to the study. Their scholastic and academic musical career ranged from 13 to 18 years. Musicians were recruited within Conservatory classes and with no monetary compensation. Singers were not recruited because of their professional vocal specialization.

40 University students (24 females, 16 males) aging on average 23 took part to the study. Participants were recruited through *Sona System* (a system for recruiting students who earn credit for their academic courses by participating in research studies). Their inclusion depended on their lack of musical studies and specific interest in music as a hobby. None of them played a musical instrument or used to listen to music for more than 1 hour per day, as ascertained by a specific questionnaire. Their scholastic and academic career ranged from 13 to 18 years.

All subjects were right-handed with normal hearing and hearing threshold. Participants with sight deficits (myopia, astigmatism, presbyopia, hyperopia) were asked to wear glasses or contact lenses in order to gain a 10/10 of acuity. None of them had never suffered from psychiatric or neurological diseases. All participants gave written and informed consent for their participation. The experiment was performed in accordance with relevant guidelines and regulations, and was approved by the ethical Committee of the University of Milano-Bicocca.

### Stimuli

Ten Italian syllables were used as stimuli, two for the training phase (/fa/ e za/) and eight for the experimental phase (see Table 4 for a description of their mechanics and place of articulation). For recording audio and video signals, a female Italian and a male Italian speakers pronounced each syllable three times. While they pronounced the 10 syllables, their face was videotaped through a fixed video camera (Samsung SMX-F30BP/EDC; 205 kbps with a sampling frequency of 48 kHz) located in front of the talker. The background behind the speaker’s face was dark (please see some example of videos provided in the supplementary files).

**Figure 4:**
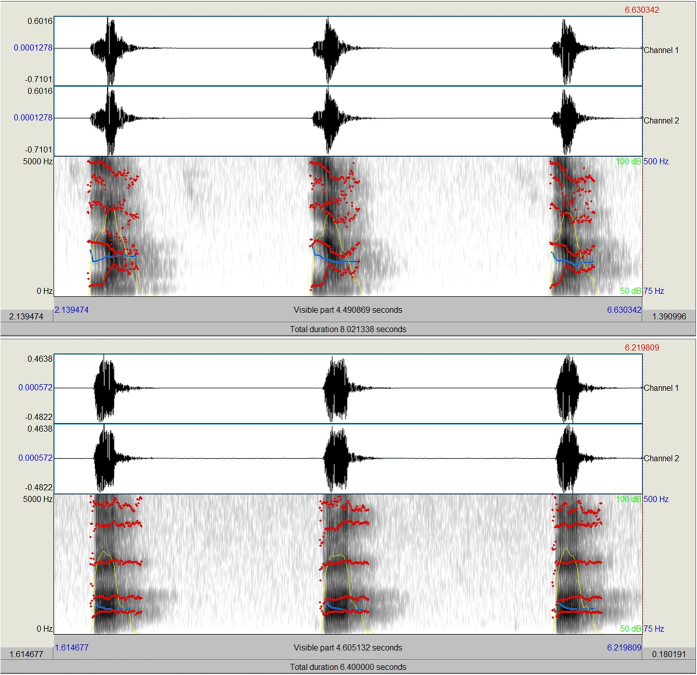
Frequency spectrum analysis relative to the phoneme /LA/ (Top) and /PA/ (bottom) obtained via *Praat* software. The spectrogram is a spectro-temporal representation of the sound. The horizontal direction of the spectrogram represents time, the vertical direction represents frequency. Darker parts of the spectrogram indicate higher energy densities, lighter parts indicate mean lower energy densities. Below each spectrogram it can be seen the frequency scale, ranging from 0 Hz to 5000 Hz.

The speakers were instructed to pronounce clearly each syllable with an interval punctuated by a metronome set at 60 BPM (beat per minute). After recording all the syllables, actors dubbed themselves by pronouncing all the incongruent phonemes while watching the mute videos. Dubbing was performed to avoid excessive adjustment in the temporal synchronization of incongruent audiovisual information. Indeed this was performed off-line via *Praat* software allowing to temporally analyze the vocal frequency spectrum (see Fig. 4).

Congruent or incongruent videos were shown via a *Powerpoint* (PPT) presentation in which they were randomly mixed in 12 different combinations casually administered to participants. To avoid an excessive time length of the experimental procedure each subject was presented with 64 videos in the McGurk condition (40 Ss), or to the unimodal auditory condition (20 Ss), or to the unimodal visual condition (20 Ss). A few minutes pause was allowed after the presentation of the 16^th^ video, while a shorter pause was allowed after the 32^th^ and the 48^th^ one.

### Procedure

The experiments were conducted at the laboratory of cognitive electrophysiology of IBFM-CNR in Milan (for controls) and at the computer lab of the Conservatory of music *Luca Marenzio* of Brescia (for musicians). Participants were randomly assigned to the McGurk audiovisual condition, in which they were instructed to report “what they had heard”, or to the auditory task, in which they listened to the MP3 sounds without watching any videos, and were asked to report “what they had heard”, or to the visual condition in which they watched the mute videos and were asked to report which syllable they thought it had been delivered. They were also instructed to maintain fixation on a red fixation circle falling on the speaker’s nose (in the McGurk condition), and to avoid any head or eye movements during the experimental session.

Each participant was provided with a pen and the preformatted response sheet. Subjects were instructed to report their response as accurately and quickly as possible on the sheet during the inter-stimulus interval (ISI). Videos were presented on a 17 inches screen placed at the height of subjects’ eyes, who were comfortably sat at a fixed distance of 80 cm from the screen. Between each video a black background was presented for 5 sec. (ISI), followed by a progressive number appearing at the center of the screen for 2 sec. It announced the next stimulus, and corresponded to its numbered response box on the sheet. The experiment was preceded by a training phase in which participants were presented with /fa/ e /za/ phonemes correctly or incorrectly paired with the labial information. The training stimuli for the unimodal tasks were unimodal and consisted in listening to or observing the pronunciation of /fa/ e /za/ phonemes.

In all conditions participants wore a set of headphones to listen to the audio and/or to avoid acoustic interferences from the outer environment.

### Data analysis

The percentage of correct responses was quantified and statistically analyzed through non parametric tests and repeated measures ANOVAs. A Wilcoxon test was applied to raw hits percentage obtained in response to each of the 8 phonemes in 3 different conditions: visual unimodal, auditory unimodal, audiovisual congruent (McGurk test).

Hit percentages were arc sin transformed in order to undergo an analyses of variance. As well known (e.g.)^73^ percentage values do not respect homoscedasticity necessary for ANOVA data distribution and for this reason need to be transformed in arcsine values. In fact the distribution of percentages is binomial while arcsine transformation of data makes the distribution normal. A mixed repeated measures ANOVA was performed on hit percentages recorded across groups and conditions (collapsed across phonemes). Its factors of variability were: one between-groups (musicians vs. controls), and one within-groups (incongruent vs. auditory condition).

Two further repeated measures ANOVAs were performed for the 2 groups to investigate in detail the effect of lip movements (visual) or phonetic (auditory) information on the McGurk effect. The dependent variable was the performance as a function of the phonemes heard or perceived. The factor of variability was “condition” whose levels were: /la/ /da/ /ta/ /ga/ /ka/ /na/ /ba/ /pa/, congruent). Tukey post-hoc test was used for comparisons among means.

## Additional Information

**How to cite this article**: Proverbio, A. M. *et al*. Skilled musicians are not subject to the McGurk effect. *Sci. Rep*. **6**, 30423; doi: 10.1038/srep30423 (2016).

## Supplementary Material

Supplementary Information

Supplementary Video S1

Supplementary Video S2

## Figures and Tables

**Table 1:** MUSICIANS: Qualitative description of auditory percepts recorded in the MGurk experiment as a function of phonetic (left) and labial (top) inputs.

		VISUAL INPUT							
		LA	DA	TA	GA	KA	NA	BA	PA
AUDITORY INPUT	LA	La = 100	La = 95Bla = 5	La = 100	La = 100	La = 100	La = 100	La = 80Pla = 15Mla = 5	La = 100
	DA	Da = 95Lda = 5	Da = 90Bda = 10	Da = 100	Da = 90Bda = 10	Da = 100	Da = 100	Da = 85Bda = 15	Da = 95Bda = 5
	TA	Ta = 100	Ta = 100	Ta = 100	Ta = 95Mta = 5	Ta = 95Lta = 5	Ta = 95Pta = 5	Ta = 90Pta = 10	Ta = 100
	GA	Ga = 90Lga = 10	Ga = 95Bka = 5	Ga = 95Dga = 5	Ga = 95Nga = 5	Ga = 95Dga = 5	Ga = 65Da = 20Pga = 5Gna = 5Pda = 5	Ga = 90Mga = 5Pga = 5	Ga = 90Ka = 5Bga = 5
	KA	Ka = 95Lka = 5	Ka = 100	Ka = 95Dka = 5	Ka = 95Tka = 5	Ka = 95Lka = 5	Ka = 95Lka = 5	Ka = 90Pka = 10	Ka = 100
	NA	Na = 100	Na = 95Mna = 5	Na = 100	Na = 100	Na = 95Mna = 5	Na = 95Mna = 5	Na = 85Mna = 10Pna = 5	Na = 100
	BA	Ba = 85Lba = 10Da = 5	Ba = 95Dba = 5	Ba = 75Da = 20Dba = 5	Ba = 100	Ba = 100	Ba = 90Bam = 10	Ba = 100	Ba = 100
	PA	Pa = 50Ta = 40Lpa = 5Lta = 5	Pa = 100	Pa = 70Ta = 20Tpa = 5Pan = 5	Pa = 70Ba = 10La = 5Pga = 5Ta = 5A = 5	Pa = 65Ta = 35	Pa = 95Lpa = 5	Pa = 100	Pa = 100

In each box the percentages (%) of musicians reporting a given percept are displayed.

**Table 2:** CONTROLS: Qualitative description of auditory percepts recorded in the MGurk experiment as a function of phonetic (left) and labial (top) inputs.

		VISUAL INPUT							
		LA	DA	TA	GA	KA	NA	BA	PA
AUDITORY INPUT	LA	La = 100	La = 95Gla = 5	La = 90Sla = 5Ta = 5	La = 100	La = 100	La = 85Mla = 5Bla = 5Ba = 5	La = 80Bla = 15Pla = 5	La = 95Bla = 5
	DA	Da = 100	Da = 100	Da = 95Sda = 5	Da = 100	Da = 95Nda = 5	Da = 95Bda = 5	Da = 85Bda = 10Ba = 5	Da = 90Ba = 5Bda = 5
	TA	Ta = 80Tam = 5Tav = 10Ka = 5	Ta = 100	Ta = 95Nta = 5	Ta = 95Pta = 5	Ta = 90Lta = 5Tla = 5	Ta = 95Tla = 5	Ta = 95Pta = 5	Ta = 100
	GA	Ga = 85Lga = 10Da = 5	Ga = 90Va = 5Pga = 5	Ga = 90Da = 5Nga = 5	Ga = 90Mga = 5Tka = 5	Ga = 90Lga = 10	Ga = 85Da = 10Na = 5	Ga = 90Mga = 5Bga = 5	Ga = 100
	KA	Ka = 90Lka = 10	Ka = 100	Ka = 95Zka = 5	Ka = 100	Ka = 100	Ka = 95Pka = 5	Ka = 95Pka = 5	Ka = 95Mka = 5
	NA	Na = 95Lna = 5	Na = 75Ma = 5Mna = 5Nga = 5	Na = 90Kna = 5Mna = 5	Na = 100	Na = 100	Na = 95Gna = 5	Na = 75Mna = 15Ba = 5Pna = 5	Na = 100
	BA	Ba = 85Lba = 5Da = 10	Ba = 100	Ba = 80Da = 20	Ba = 100	Ba = 80Ta = 5Gba = 5Gda = 5Da = 5	Ba = 100	Ba = 95Bam = 5	Ba = 100
	PA	Pa = 50Ta = 30Ra = 5Lta = 10A = 5	Pa = 100	Pa = 75Ta = 15Tpa = 10	Pa = 35A = 45Ta = 15Da = 5	Pa = 35Ta = 45Da = 5Lta = 10Tan = 5	Pa = 80Ka = 15Pla = 5	Pa = 95Pam = 5	Pa = 100

As previously reported by studies on Italian speakers (D’Ausilio *et al*.^10^ or Bovo and coworkers)^11^ McGurk illusions were more frequently found when bilabial phonemes were presented acoustically (e.g.: /PA/) and non-labials (/GA/ or /KA/) visually.

**Table 3:** Musicians of the *Luca Marenzio Conservatory of Music in Brescia* participating to the study, in the various conditions.

MUSICIANS						
*Ss*	*Sex*	*Age*	*YMS*	*AoA*	*Degree*	*Instrument*
1	M	21	13	6	M.A.	Cello
2	F	23	13	8	B.A.	Cello
3	M	20	13	5	M.A. near-graduate	Clarinet
4	M	19	13	9	M.A.	Clarinet
5	M	28	13	15	B.A.	Contrabass
6	F	24	13	4	B.A.	Flute
7	F	21	13	8	B.A.	Flute
8	F	21	13	8	M.A.	Flute
9	F	24	13	6	M.A. near-graduate	Flute
10	M	19	13	5	Near-graduate	Flute
11	F	19	13	6	B.A.	Flute
12	F	20	13	8	Near-graduate	Flute
13	F	27	16	7	M.A.	Flute
14	F	22	13	6	B.A.	Harp
15	M	19	13	10	Near-graduate	Horn
16	F	28	16	7	M.A. near-graduate	Mandolin
17	M	23	13	8	B.A.	Oboe
18	M	26	18	9	Near-graduate	Percussion
19	F	25	16	10	M.A.	Percussion
20	M	21	13	18	B.A.	Percussion
21	M	32	13	11	M.A. near-graduate	Piano
22	M	26	13	11	M.A. near-graduate	Piano
23	M	32	13	8	M.A. near-graduate	Piano
24	M	21	13	7	Near-graduate	Piano
25	M	22	13	6	B.A.	Piano
26	M	24	13	4	M.A.	Piano
27	M	19	13	6	Near-graduate	Piano
28	M	24	13	12	B.A.	Saxophone
29	M	21	13	13	B.A.	Saxophone
30	M	32	13	7	M.A. near-graduate	Saxophone
31	M	29	13	12	B.A.	Trombone
32	M	19	13	10	Near-graduate	Trumpet
33	M	21	13	4	M.A.	Trumpet
34	F	22	13	6	B.A.	Viola
35	F	20	13	6	B.A.	Viola
36	M	20	13	6	B.A.	Violin
37	M	26	13	8	B.A.	Violin
38	F	25	13	6	M.A. near-graduate	Violin
39	M	28	13	12	M.A.	Violin
40	F	22	13	7	B.A.	Violin

Musicians YMS = length of musical studies (in years), AoA = age of acquisition, M.B.A = Instrument = musical instrument mainly played.

**Table 4:** List of phonemes used in the study, as a function of their phonetic and articulatory characteristics.

	Articulatory place							
	Bilabial		Dental		Alveolar		Velar	
Articulatory mode/ Sonority	**Unv**	**Voi**	**Unv**	**Voi**	**Unv**	**Voi**	**Unv**	**Voi**
Occlusive	/pa/	/ba/	/ta/	/da/			/ka/	/ga/
Nasal						/na/		
Lateral						/la/		

Unv = unvoiced; Voi = Voiced.

## References

[b1] McGurk H. & MacDonald J. Hearing lips and seeing voices. Nature 264, 746–748 (1976). 1012311 10.1038/264746a0

[b2] Tiippana K. What is the McGurk effect? Front Psychol 5, 725 (2014). 25071686 10.3389/fpsyg.2014.00725 PMC4091305

[b3] Moro S. & Steeves J. Audiovisual integration in people with one eye: Normal temporal binding window and sound induced flash illusion but reduced McGurk effect. J Vis. 15(12), 721 (2015).

[b4] White T. P. . Eluding the illusion? Schizophrenia, dopamine and the McGurk effect. Front Hum Neurosci. 5(8), 565 (2014). 25140138 10.3389/fnhum.2014.00565 PMC4122162

[b5] Setti A., Burke K. E., Kenny R. & Newell F. N. Susceptibility to a multisensory speech illusion in older persons is driven by perceptual processes. Front Psychol 4, 575 (2013). 24027544 10.3389/fpsyg.2013.00575 PMC3760087

[b6] Pearl D. . Differences in audiovisual integration, as measured by McGurk phenomenon, among adult and adolescent patients with schizophrenia and age-matched healthy control groups. Compr Psychiatry 50, 186–192 (2009). 19216897 10.1016/j.comppsych.2008.06.004

[b7] Proverbio A. M. & Zani A. Developmental changes in the linguistic brain after puberty. Trends Cogn Sci. 9(4), 164–167 (2005). 15808495 10.1016/j.tics.2005.02.001

[b8] Nath A. R. & Beauchamp M. S. A neural basis for interindividual differences in the McGurk effect, a multisensory speech illusion. Neuroimage 59(1), 781–787 (2012). 21787869 10.1016/j.neuroimage.2011.07.024 PMC3196040

[b9] Gentilucci M. & Cattaneo L. Automatic audiovisual integration in speech perception. Exp Brain Res. 167(1), 66–75 (2005). 16034571 10.1007/s00221-005-0008-z

[b10] D’Ausilio A., Bartoli E., Maffongelli L., Berry J. J. & Fadiga L. Vision of tongue movements bias auditory speech perception. Neuropsychologia 63, 85–91 (2014). 25172391 10.1016/j.neuropsychologia.2014.08.018

[b11] Bovo R., Ciorba A., Prosser S. & Martini A. The McGurk phenomenon in Italian listeners. Acta Otorhinolaryngol Ital 29(4), 203–208 (2009). 20161878 PMC2816368

[b12] Tervaniemi M. Musicians—Same or Different? Ann NY Acad Sci. 1169(1), 151–156 (2009). 19673771 10.1111/j.1749-6632.2009.04591.x

[b13] Schlaug G. The brain of musicians. A model for functional and structural adaptation. Ann NY Acad Sci. 930, 281–299 (2001). 11458836

[b14] Bengtsson S. L. . Extensive piano practicing has regionally specific effects on white matter development, Nature Neurosci. 8, 1148–1150 (2005). 16116456 10.1038/nn1516

[b15] Jäncke L. The plastic brain. Restor Neurol Neurosci. 27(5), 521–538 (2009). 19847074 10.3233/RNN-2009-0519

[b16] Rüber T., Lindenberg R. & Schlaug G. Differential Adaptation of Descending Motor Tracts in Musicians. Cereb Cortex 25(6), 1490 (2015). 24363265 10.1093/cercor/bht331 PMC4428294

[b17] Zatorre R. J., Chen J. L. & Penhune V. B. When the brain plays music: auditory-motor interactions in music perception and production. Nature Rev Neurosci. 8(7), 547–558 (2007). 17585307 10.1038/nrn2152

[b18] Proverbio A. M., Manfredi M., Zani A. & Adorni R. Musical expertise affects neural bases of letter recognition. Neuropsychologia 51(3), 538–549 (2013). 23238370 10.1016/j.neuropsychologia.2012.12.001

[b19] Gaser C. & Schlaugh G. Brain structures differ between musicians and non-musicians. J Neurosci. 23, 9240–9245 (2003). 14534258 10.1523/JNEUROSCI.23-27-09240.2003 PMC6740845

[b20] Harris R. & De Jong B. M. Cerebral activations related to audition-driven performance imagery in professional musicians. PLos One 8, 9(4) (2014). 10.1371/journal.pone.0093681 PMC3979724 24714661

[b21] Lee D. J., Chen Y. & Schlaug G. Corpus callosum: musician and gender effects. Neuroreport 10,14(2), 205–209 (2003). 12598730 10.1097/00001756-200302100-00009

[b22] Oztürk A. H., Tasçioglu B., Aktekin M., Kurtoglu Z. & Erden I. Morphometric comparison of the human corpus callosum in professional musicians and non-musicians by using *in vivo* magnetic resonance imaging. J Neuroradiol 29(1), 29–34 (2002). 11984475

[b23] Elmer S. . Music and Language Expertise Influence the Categorization of Speech and Musical Sounds: Behavioural and Electrophysiological Measurements. J Cogn Neurosci. 26(10), 2356–2369 (2014). 24702451 10.1162/jocn_a_00632

[b24] Elmer S., Meyer M. & Jäncke L. Neurofunctional and behavioural correlates of phonetic and temporal categorization in musically trained and untrained subjects. Cerebral Cortex 22, 650–658 (2012). 21680844 10.1093/cercor/bhr142

[b25] Chobert J., Marie C., François C., Schön D. & Besson M. Enhanced passive and active processing of syllables in musician children. J Cogn Neurosci. 23(12), 3874–3887 (2011). 21736456 10.1162/jocn_a_00088

[b26] Chobert J., François C., Velay J. L. & Besson M. Twelve months of active musical training in 8- to 10-year-old children enhances the preattentive processing of syllabic duration and voice onset time. Cereb Cortex 24(4), 956–967 (2014). 23236208 10.1093/cercor/bhs377

[b27] Kühnis J., Elmer S., Meyer M. & Jäncke L. Musicianship boosts perceptual learning of pseudoword-chimeras: an electrophysiological approach. Brain Topography 26(1), 110–125 (2013). 22736323 10.1007/s10548-012-0237-y

[b28] Kühnis J., Elmer S. & Jäncke L. Auditory Evoked Responses in Musicians during Passive Vowel Listening Are Modulated by Functional Connectivity between Bilateral Auditory-related Brain Regions. J Cognitive Neurosci. 4, 1–12 (2014). 10.1162/jocn_a_00674 24893742

[b29] Gaab N. . Neural correlates of rapid spectrotemporal processing in musicians and nonmusicians, Ann NY Acad Sci. 1060, 82–88 (2005). 16597753 10.1196/annals.1360.040

[b30] Parbery-Clark A., Tierney A., Strait D. L. & Kraus N. Musicians have fine-tuned neural distinction of speech syllables. Neuroscience 6(219), 111–119 (2012). 22634507 10.1016/j.neuroscience.2012.05.042 PMC3402586

[b31] Strait D. L. & Kraus N. Can you hear me now? Musical training shapes functional brain networks for selective auditory attention and hearing speech in noise. Front Psychol 13, 2:113 (2011). 21716636 10.3389/fpsyg.2011.00113 PMC3115514

[b32] Kraus N. & Chandrasekaran B. Music training for the development of auditory skills, Nat Rev Neurosci. 11(8), 599–605 (2010). 20648064 10.1038/nrn2882

[b33] Baumann S. . A network for audio-motor coordination in skilled pianists and non-musicians. Brain Res. 1161, 65–78 (2007). 17603027 10.1016/j.brainres.2007.05.045

[b34] Meyer M., Elmer S. & Jäncke L. Musical expertise induces neuroplasticity of the planum temporale. Ann NY Acad Sci. 1252, 116–123 (2012). 22524348 10.1111/j.1749-6632.2012.06450.x

[b35] Proverbio A. M., Calbi M., Manfredi M. & Zani A. Audio-visuomotor processing in the Musician’s brain: an ERP study on professional violinists and clarinetists. Sci Reports 4, 5866 (2014). 10.1038/srep05866 PMC5376193 25070060

[b36] Proverbio A. M., Attardo L., Cozzi M. & Zani A. The effect of musical practice on gesture/sound pairing. Front Psychol: Audit Cogn Neurosci. 6, 2;6:376 (2015). 10.3389/fpsyg.2015.00376 PMC4382982 25883580

[b37] Proverbio A. M. & Orlandi A. Instrument-Specific Effects of Musical Expertise on Audiovisual Processing (Clarinet versus Violin), Psychology of Music 33(4) (2016).

[b38] Patel A. D. Why would musical training benefit the neural encoding of speech? The OPERA hypothesis. Front Psychol 2, 142 (2011). 21747773 10.3389/fpsyg.2011.00142 PMC3128244

[b39] Lee H. L. H. & Noppeney U. Long-term music training tunes how the brain temporally binds signals from multiple senses. Pnas 108(51), E1441–E1450 (2011). 22114191 10.1073/pnas.1115267108 PMC3251069

[b40] Paraskevopoulos E., Kuchenbuch A., Herholz S. C. & Pantev C. Musical expertise induces audiovisual integration of abstract congruency rules. J Neurosci. 32(50), 18196–18203 (2012). 23238733 10.1523/JNEUROSCI.1947-12.2012 PMC6621720

[b41] Massaro D. W. Perceiving Talking Faces. Cambridge, MA (MIT Press 1998).

[b42] Sekiyama K. & Tohkura Y. McGurk effect in non-English listeners: few visual effects for Japanese subjects hearing Japanese syllables of high auditory intelligibility. J. Acoust Soc Am. 90, 1797–1805 (1991). 1960275 10.1121/1.401660

[b43] Green K. P. & Norrix L. W. Acoustic cues to place of articulation and the McGurk effect: the role of release bursts, aspiration, and formant transitions. J Speech Lang Hear Res. 40, 646–665 (1997). 9210121 10.1044/jslhr.4003.646

[b44] Jiang J. & Bernstein L. E. Psychophysics of the McGurk and other audiovisual speech integration effects. J Exp Psychol Hum Percept Perform 37, 1193–1209 (2011). 21574741 10.1037/a0023100 PMC3149717

[b45] Tiippana K., Andersen T. S. & Sams M. Visual attention modulates audiovisual speech perception. Eur J Cogn Psychol 16, 457–472 (2004).

[b46] Gurler D., Doyle N., Walker E., Magnotti J. & Beauchamp M. A link between individual differences in multisensory speech perception and eye movements. Atten Percept Psychophys 77(4), 1333–1341 (2015). 25810157 10.3758/s13414-014-0821-1 PMC4437244

[b47] Bernstein L. E., Demorest M. E. & Tucker P. E. Speech perception without hearing. Percept Psychophys 62, 233–252 (2000). 10723205 10.3758/bf03205546

[b48] Bernstein L. E. & Liebenthal E. Neural pathways for visual speech perception. Front Neurosci. 1(**8**), 386 (2014). 25520611 10.3389/fnins.2014.00386 PMC4248808

[b49] Calvert G. A. . Response amplification in sensory-specific cortices during crossmodal binding. Neuroreport 10, 2619–2623 (1999). 10574380 10.1097/00001756-199908200-00033

[b50] Calvert G. A., Campbell R. & Brammer M. J. Evidence from functional magnetic resonance imaging of crossmodal binding in the human heteromodal cortex. Curr Biol. 10, 649–657 (2000). 10837246 10.1016/s0960-9822(00)00513-3

[b51] Calvert G. A. & Campbell R. Reading speech from still and moving faces: the neural substrates of visible speech. J Cogn Neurosci. 15, 57–70 (2003). 12590843 10.1162/089892903321107828

[b52] Massaro D. W., Cohen M. M., Tabain M. & Beskow J. Animated speech: research progress and applications, In Audiovisual Speech Processing (eds Clark R. B., Perrier J. P. & Vatikiotis-Bateson E.) 246–272 (Cambridge: Cambridge University 2012).

[b53] Callan D. E., Jones J. A. & Callan A. Multisensory and modality specific processing of visual speech in different regions of the premotor cortex. Front Psychol 5, 389 (2014). 24860526 10.3389/fpsyg.2014.00389 PMC4017150

[b54] Calvert G. A. Crossmodal processing in the human brain: insights from functional neuroimaging studies. Cereb Cortex 11, 1110–1123 (2001). 11709482 10.1093/cercor/11.12.1110

[b55] Calvert G. A. . Activation of auditory cortex during silent lipreading. Science 276, 593–596 (1997). 9110978 10.1126/science.276.5312.593

[b56] Pekkola J. . Primary auditory cortex activation by visual speech: an fMRI study at 3 T. Neuroreport 8,16(2), 125–128 (2005). 15671860 10.1097/00001756-200502080-00010

[b57] Sams M. . Seeing speech: visual information from lip movements modifies activity in the human auditory cortex. Neurosci Lett. 127, 141–145 (1991). 1881611 10.1016/0304-3940(91)90914-f

[b58] Varnet L., Wang T., Peter C., Meunier F. & Hoen M. How musical expertise shapes speech perception: evidence from auditory classification images. Sci Rep. 24(5), 14489 (2015). 26399909 10.1038/srep14489 PMC4585866

[b59] Slater J. . Music training improves speech-in-noise perception: Longitudinal evidence from a community-based music rogram. Behav Brain Res. 291, 244–252 (2105). 26005127 10.1016/j.bbr.2015.05.026

[b60] Strait D. L., Parbery-Clark A., O’Connell S. & Kraus N. Biological impact of preschool music classes on processing speech in noise. Dev Cogn Neurosci. 6, 51–60 (2013). 23872199 10.1016/j.dcn.2013.06.003 PMC3844086

[b61] Parbery-Clark A., Skoe E. & Kraus N. Musical experience limits the degradative effects of background noise on the neural processing of sound. J Neurosci. 29, 14100–14107 (2009). 19906958 10.1523/JNEUROSCI.3256-09.2009 PMC6665054

[b62] Zendel B. R., Tremblay C. D., Belleville S. & Peretz I. The impact of musicianship on the cortical mechanisms related to separating speech from background noise, J Cogn Neurosci. 27, 1044–1059 (2015). 25390195 10.1162/jocn_a_00758

[b63] Paraskevopoulos E., Kraneburg A., Herholz S. C., Bamidis P. D. & Pantev C. Musical expertise is related to altered functional connectivity during audiovisual integration. Pnas, 112(40), 12522–12527 (2015). 26371305 10.1073/pnas.1510662112 PMC4603494

[b64] Parkinson A. L. . Effective connectivity associated with auditory error detection in musicians with absolute pitch. Front Neurosci. 5, 8:46 (2014). 24634644 10.3389/fnins.2014.00046 PMC3942878

[b65] Lotze M., Scheler G., Tan H.-R. M., Braun C. & Birbaumer N. The musician’s brain: functional imaging of amateurs and professionals during performance and imagery. Neuroimage 20(3), 1817–1829 (2003). 14642491 10.1016/j.neuroimage.2003.07.018

[b66] Nath A. R. & Beauchamp M. S. Dynamic changes in superior temporal sulcus connectivity during perception of noisy audiovisual speech. Journal of Neuroscience, 31, 1704–1714 (2011). 21289179 10.1523/JNEUROSCI.4853-10.2011 PMC3050590

[b67] Nath A. R., Fava E. E. & Beauchamp M. S. Neural correlates of interindividual differences in children’s audiovisual speech perception. J Neurosci. 31(39), 13963–13971 (2011). 21957257 10.1523/JNEUROSCI.2605-11.2011 PMC3203203

[b68] Beauchamp M. S., Nath A. R. & Pasalar S. fMRI-guided transcranial magnetic stimulation reveals that the superior temporal sulcus is a cortical locus of the McGurk effect. J Neurosci. 30, 2414–2417 (2010). 20164324 10.1523/JNEUROSCI.4865-09.2010 PMC2844713

[b69] Nikjeh D. A., Lister J. J. & Frisch S. A. Preattentive cortical-evoked responses to pure tones, harmonic tones, and speech: influence of music training. Ear Hear 30(4), 432–446 (2009). 19494778 10.1097/AUD.0b013e3181a61bf2

[b70] Micheyl C., Delhommeau K., Perrot X. & Oxenham A. J. Influence of musical and psychoacoustical training on pitch discrimination. Hear Res. 219(**1–2**), 36–47 (2006). 16839723 10.1016/j.heares.2006.05.004

[b71] Herholz S. C., Lappe C. & Pantev C. Looking for a pattern: an MEG study on the abstract mismatch negativity in musicians and nonmusicians. BMC Neurosci. 10, 42 (2009). 19405970 10.1186/1471-2202-10-42 PMC2683848

[b72] Fujioka T., Trainor L. J., Ross B., Kakigi R. & Pantev C. Musical training enhances automatic encoding of melodic contour and interval structure. J Cogn Neurosci. 16, 1010–1021 (2004). 15298788 10.1162/0898929041502706

[b73] Snedecor G. W. & Cochran W. G. Statistical Methods (8^th^ ed.) (Iowa State: University Press, 1989).

