# Supplementary material for: Skilled musicians are not subject to the McGurk effect
Source: Sci Rep. 2016 Jul 25;6:30423. doi: 10.1038/srep30423 (PMC4958963; doi:10.1038/srep30423)
Supplement: Supplementary Information [file srep30423-s1.pdf]

Skilled musicians are not subject to the McGurk effect

Alice M. Proverbio[1*], Gemma Massetti[1], Ezia Rizzi[1,2], Alberto Zani[2]

[1]*Milan-Mi Center for Neuroscience, Dept. of Psychology, University of Milano-Bicocca*

[2]*IBFM-CNR, Milan, Italy*

Supplementary files. Example of video used as stimuli

1) McGurk condition: see /KA/, hear /KA/.

2) McGurk condition: see /KA/, hear/PA/, understand /TA/ .
